# Supplementary material for: Structural basis for the E3 ligase activity enhancement of yeast Nse2 by SUMO-interacting motifs
Source: Nat Commun. 2021 Dec 1;12:7013. doi: 10.1038/s41467-021-27301-9 (PMC8636563; doi:10.1038/s41467-021-27301-9)
Supplement: Supplementary file 1 — Supplementary Information [file 41467_2021_27301_MOESM1_ESM.pdf]

# Supplementary information

## Structural basis for the E3 ligase enhancement of yeast Nse2 by SUMO-Interacting Motifs

Nathalia Varejão[1], Jara Lascorz[1], Joan Codina-Fabra[2], Gemma Bellí[2], Helena Borras[1], Jordi Torres-Rosell[2] and David Reverter[1*]

[1]Institut de Biotecnologia i de Biomedicina (IBB) and Dept. de Bioquímica i Biologia Molecular, Universitat Autònoma de Barcelona, 08193 Bellaterra, Spain.
[2]IRBLLEIDA, Dept. Ciències Mèdiques Bàsiques, Universitat de Lleida, Lleida, Spain.
These authors contributed equally: Nathalia Varejão, Jara Lascorz
*Correspondence should be addressed to David Reverter (email: david.reverter@uab.cat)

Supplementary information Inventory:
Supplementary Tables 1-2
Supplementary Figures 1-7

**Supplementary Table 1. Crystallographic statistics of Nse2/Smc5 in complex with the E2-SUMO thioester mimetic and the backside $SUMO_B$.**

| Data collection | Nse2/Smc5 – Ubc9-$Smt3_D$ – $Smt3_B$ |
|---|---|
| Beamline | ALBA-XALOC |
| Space group | $P\ 2_12_12_1$ |
| Wavelength (Å) | 0.9792 |
| Resolution (Å) | 47.14-3.31 (3.58-3.31) |
| a, b, c (Å) | 71,14, 103.24, 115.61 |
| α, β, γ (°) | α = β = γ = 90 |
| Unique reflections | 13097 |
| Data redundancy | 5.4 (5.5) |
| $R_{merge}$ | 0.13 (0.95) |
| CC (1/2) | 0.99 (0.68) |
| I/σ | 9.7 (2.0) |
| Completeness (%) | 99.2 (96.7) |
| Refinement | |
| Resolution (Å) | 47.13 - 3.31 |
| Non-anomalous reflections | 13056 |
| $R_{work}/R_{free}$ | 0.235 / 0.293 |
| Number of all atoms | 4561 |
| RMSD bond (Å)/Angle (°) | 0.009 / 1.517 |
| Ramachandran plot | |
| Favored (%) | 87.77 |
| Allowed (%) | 10.40 |
| Disallowed (%) | 1.81 |

**Supplementary Table 2 – List of the used primers.**

| Primer Name | | Sequence (5'-3') |
|---|---|---|
| **Smc5** | | |
| Arm/SMC5 | Forward | CGCGGGATCCGATAAAAAACCATTTGC |
| | Reverse | GCCTGTCGACTTATTGGCTCTTCAAATCAGCTTC |
| shortArm/SMC5 | Forward | GGACCCATGGGCACTGATGAGTTCCTGAAAGC |
| | Reverse | GGACCTCGAGTTACTTCTGACAATTCTTTAAAC |
| FLNse2 | Forward | CAGTCTCGAGGCCTTGAACGATAATCCTATACC |
| | Reverse | GCTGGGATCCTTATAAAACATCGATGGCTTGAC |
| shortNse2,Δ26 | Forward | GCTGCTCGAGGCCCGAGACTTATCAAATATATATC |
| | Reverse | GCTG GGATCC TTA TAAAACATCGATGGCTTGAC |
| shortNse2, ΔN26/Δ83-134 | Forward | GGACGGTACCGCAACCATGGTTAATAACACAGATAC |
| | Reverse | ACGCGGTACCTGAGTTGGATTCCGATTCG |
| CtΔ16Nse2 | Forward | GATCGCCAAGATGAAATAATCTCAGGAACAGG |
| | Reverse | CCTGTTCCTGAGATTATTTCATCTTGGCGATC |
| CtΔ8Nse2 | Forward | GAACAGGATAAAAGATGAAGTCAAGCCATC |
| | Reverse | GATGGCTTGACTTCATCTTTTATCCTGTTC |
| CtΔ4Nse2 | Forward | AGTAGTCAAGCCTAGGATGTTTTATGA |
| | Reverse | TCATAAAACATCCTAGGCTTGACTACT |
| I264A/V266ANse2 | Forward | AGTAGTCAAGCCGCCGATGCTTTATGACTCGAGCGC |
| | Reverse | GCGCTCGAGTCATAAAGCATCCGGGGCTTGACTACT |
| I264PNse2 | Forward | AGTAGTCAAGCCCCCGATGTTTTATGA |
| | Reverse | TCATAAAACATCGGGGGCTTGACTACT |
| V266RNse2 | Forward | CAAGCCATCGATCGTTTATGACTCGAG |
| | Reverse | CTCGAGTCATAAACGATCGATGGCTTG |
| S260ENse2 | Forward | GAACAGGATAAAAGAGAGAGTCAAGCCATCGAT |
| | Reverse | ATCGATGGCTTGACTCTCTCTTTTATCCTGTTC |
| S261ENse2 | Forward | CAGGATAAAAGAAGTGAGCAAGCCATCGATGTT |
| | Reverse | AACATCGATGGCTTGCTCACTTCTTTTATCCTG |
| S260E/S261ENse2 | Forward | CAGGATAAAAGAGAAGAGCAAGCCATCGATGTT |
| | Reverse | AACATCGATGGCTTGCTCTTCTCTTTTATCCTG |
| Δ160-176Nse2 | Forward | GCCTGGTACCGGTGGTAAAATTGAATTGACTTGTCC |
| | Reverse | GCCTGGTACCTACACAAGTTGGATCATTCCA |
| G177PNse2 | Forward | CTACAAATAGAACCCGGGAAAATTGAATTG |
| | Reverse | CAATTCAATTTTCCCGGGTTCTATTTGTAG |
| E170R/D171R/D172RNse2 | Forward | CTGCAAAACCCCGCAGACAGAAGAAGACTGCAGATAGAAGGTGG |
| | Reverse | CCACCTTCTATCTGCAGTCTTCTTCTGTCTGCGGGGTTTTGCAG |
| Nse2nonstop·Smt3ΔGG | Forward (Nse2) | CAGT CTCGAG GCCTTGAACGATAATCCTATACC |
| | Reverse (Nse2) | GCTGGGATCCTAAAACATCGATGGCTTGAC |
| | Forward (FLSmt3) | GCTGGGATCCTCGGACTCAGAAGTCAATC |
| | Forward (Δ18Smt3) | GCTGGGATCCGCCTGAGACTCACATCAATTTAAAGG |
| | Reverse (FLandΔ18Smt3) | GCTGGGATCCTTAAATCTGTTCTCTGTGAGCCTC |
| Ubc9 A129K | Forward | AATCCAAATTCCCCTAAGCAAGAGCCTGCATGG |
| | Reverse | CCATGCAGGCTCTTGCTTAGGGGAATTTGGATT |
| Ubc9 K153R | Forward | GTTTTGCTTCAAGCTAGACAGTACTCTAAA |
| | Reverse | TTTAGAGTACTGTCTAGCTTGAAGCAAAAC |
| K11CSmt3 | Forward | GTCAATCAAGAAGCTTGCCCAGAGGTCAAGCCAG |
| | Reverse | CTGGCTTGACCTCTGGGCAAGCTTCTTGATTGAC |
| D68RSmt3 | Forward | CCTTAAGATTCTTGTACCGCGGTATTAGAATTCAAGC |
| | Reverse | GCTTGAATTCTAATACCGCGGTACAAGAATCTTAAGG |

a

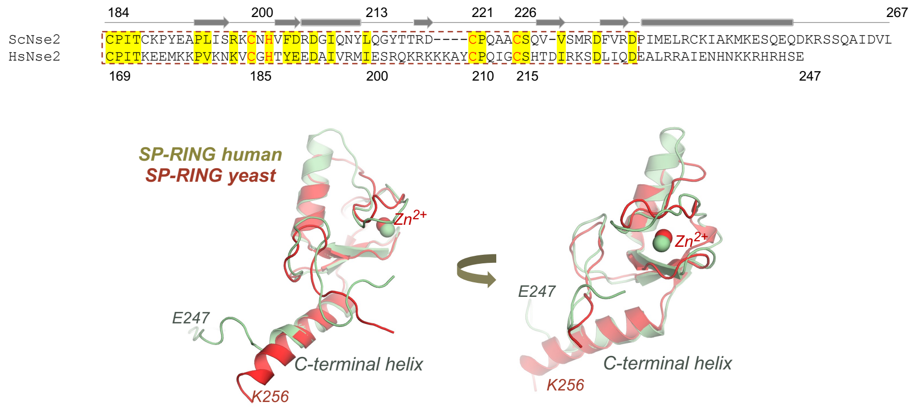


b

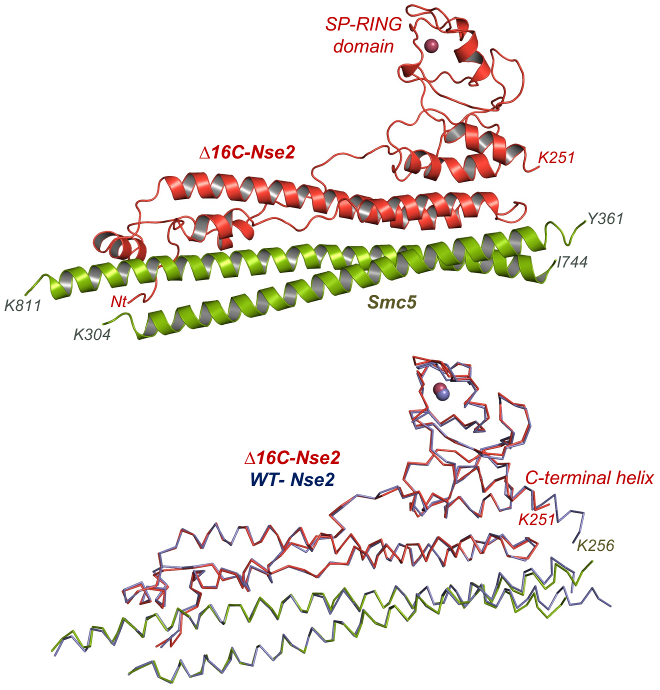


| | Δ16-Nse2/Smc5 |
|---|---|
| **Data collection** | |
| Space group | $C222_1$ |
| Unit cell parameters (Å) | 91.11, 47.43, 250.12 |
| Wavelength (nm) | 0.9791 |
| Resolution range (Å) | 45.6 - 3.29 |
| Rmerge | 0.09 (0.84) |
| Rpim | 0.04 (0.33) |
| (I/σ(I)) | 11.5 (2.0) |
| Completeness (%) | 98.1 (100) |
| Multiplicity | 6.6 (7.2) |
| CC (1/2) | 0.99 (0.72) |
| **Structure refinement** | |
| Resolution range (Å) | 45.5 - 3.3 |
| No. of unique reflections | 8,362 |
| $R_{work}/R_{free}$ (%) | 0.23/0.33 |
| No. of atoms | |
| Protein | 3,010 |
| $Zn^{2+}$ | 1 |
| Overall B factors ($Å^2$) | 102.48 |
| R.m.s. deviations | |
| Bonds (Å) | 0.012 |
| Angles (°) | 1.42 |

c

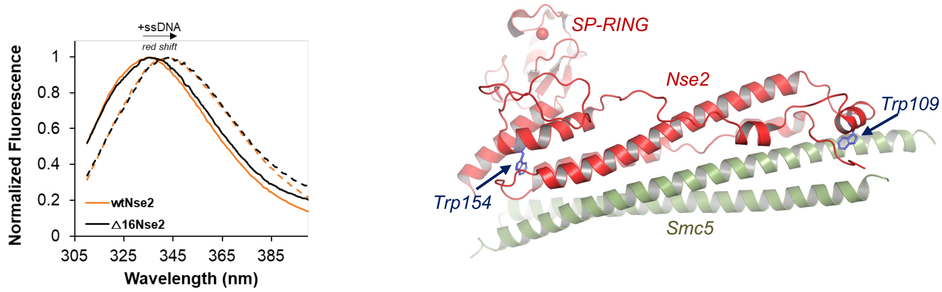


**Supplementary Figure 1. Comparisons between yeast and human Nse2. a** Pair-wise alignment of Nse2 sequences from *Saccharomyces cerevisiae* and *Homo sapiens* and superimposition of their SP-RING domains (PDBs 3HTK and 2YU4) revealing different lengths of the C-terminal α-helix, and absence of C-terminal tail on the human protein. **b** Crystal structure of yeast Δ16-Nse2/Smc5 (cartoon representation). Superimposition of the Δ16-Nse2/Smc5 and wild-type (PDB 3HTK) showing correct folding and assembly of the former. Table summarizing data-collection and refinement statistics. **c** Intrinsic Trp-fluorescence from Δ16-Nse2/Smc5 and wild-type spectra highlighting similar emission values and DNA-dependent red-shift. Cartoon representation of the Δ16-Nse2/Smc5, showing the position of the two tryptophan residues present in the complex.

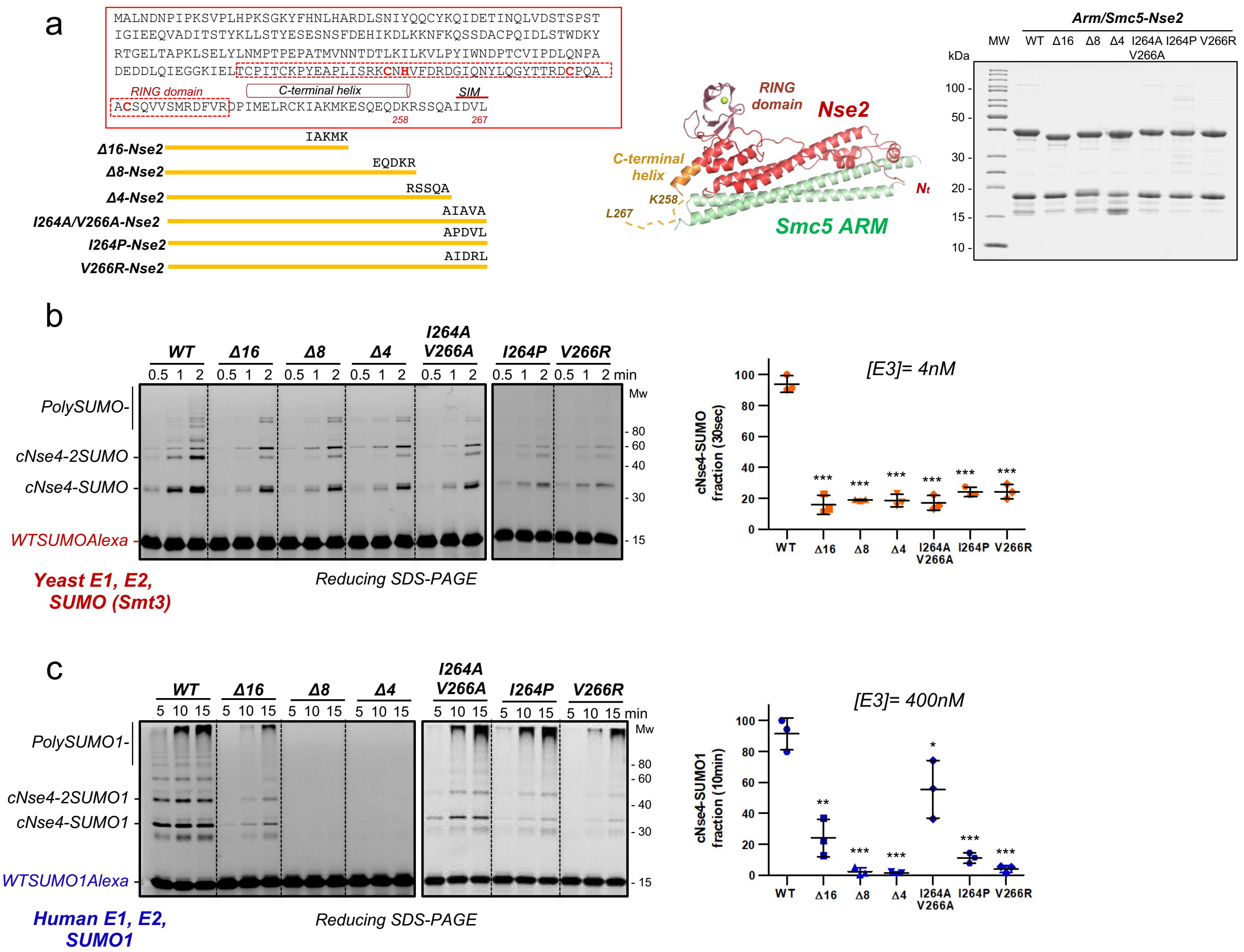


**Supplementary Figure 2. Comparisons between yeast and human Nse2. a** Sequence of Nse2 from *Saccharomyces cerevisiae*, labeling SP-RING domain, C-terminal α-helix and SIM2. Schematic representation of C-terminal deletions and mutants (*left*). Cartoon representation of the structure of wild-type Nse2/Smc5 (PDB 3HTK, *middle*). SDS-PAGE of the purified proteins (*right*). **b** Multiple-turnover SUMOylation reactions of yeast Nse2/Smc5 complex (wild-type and mutants) using cNse4 substrate at 30ºC (*left*). Bar graphs showing that point mutations or deletion of SIM2 reduces E3 activity (*right*). The quantified rate data show mean ± s.d. (n = 3 technical replicates). All experiments were performed in the presence of 50nt ssDNA. **c** Multiple-turnover SUMOylation reactions of yeast Nse2/Smc5 complex using human E1, E2, SUMO1, and cNse4 substrate at 30ºC (*left*). Error bars showing that point mutations or deletion of SIM2 reduce E3 activity (right). Data values represent mean ±SD, n = 3 technical replicates. Significance was measured by a two-tailed unpaired t-test relative to wild-type. **P* < 0.05, ***P* < 0.01, ****P* < 0.001. Exact *P* values from the left to right: **b** 0.0001, 0.0001, 0.0001, 0.0001, 0.0001, 0.0001; **c** 0.0019, 0.0001, 0.0001, 0.0438, 0.0002, 0.0001. Source data are provided as a Source Data file. All experiments were performed in the presence of 50nt ssDNA.

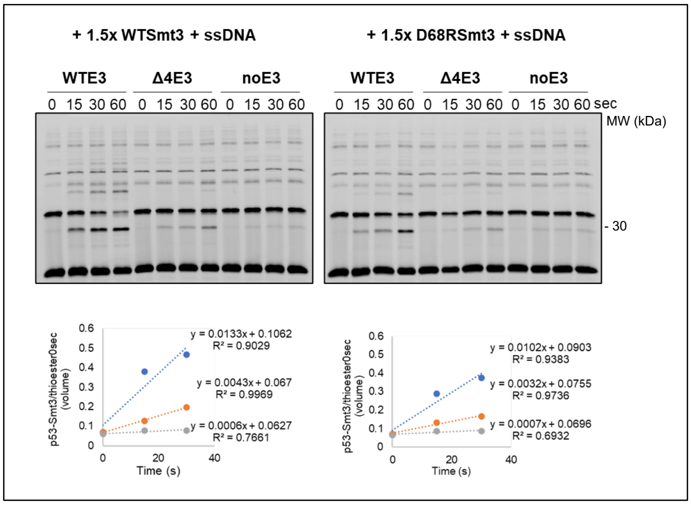


**Supplementary Figure 3. Raw data of SUMOylation reactions of Nse2/Smc5 complex using Ubc9~$^{D68R}$Smt3 thioester in the presence of extra wild-type or D68R Smt3.** a Representative SDS-PAGE of the single-turnover SUMOylation reactions using increasing concentrations of $_{c}$p53 (only 2 μM is shown), Ubc9~$^{D68R}$Smt3-Alexa488 thioester stopped with EDTA, 50 nt ssDNA, yeast Nse2/Smc5 complex (WT and Δ4SIM2) in the presence of non-conjugatable extra wild-type or D68R Smt3. Line graph showing results of band densitometry measurements.

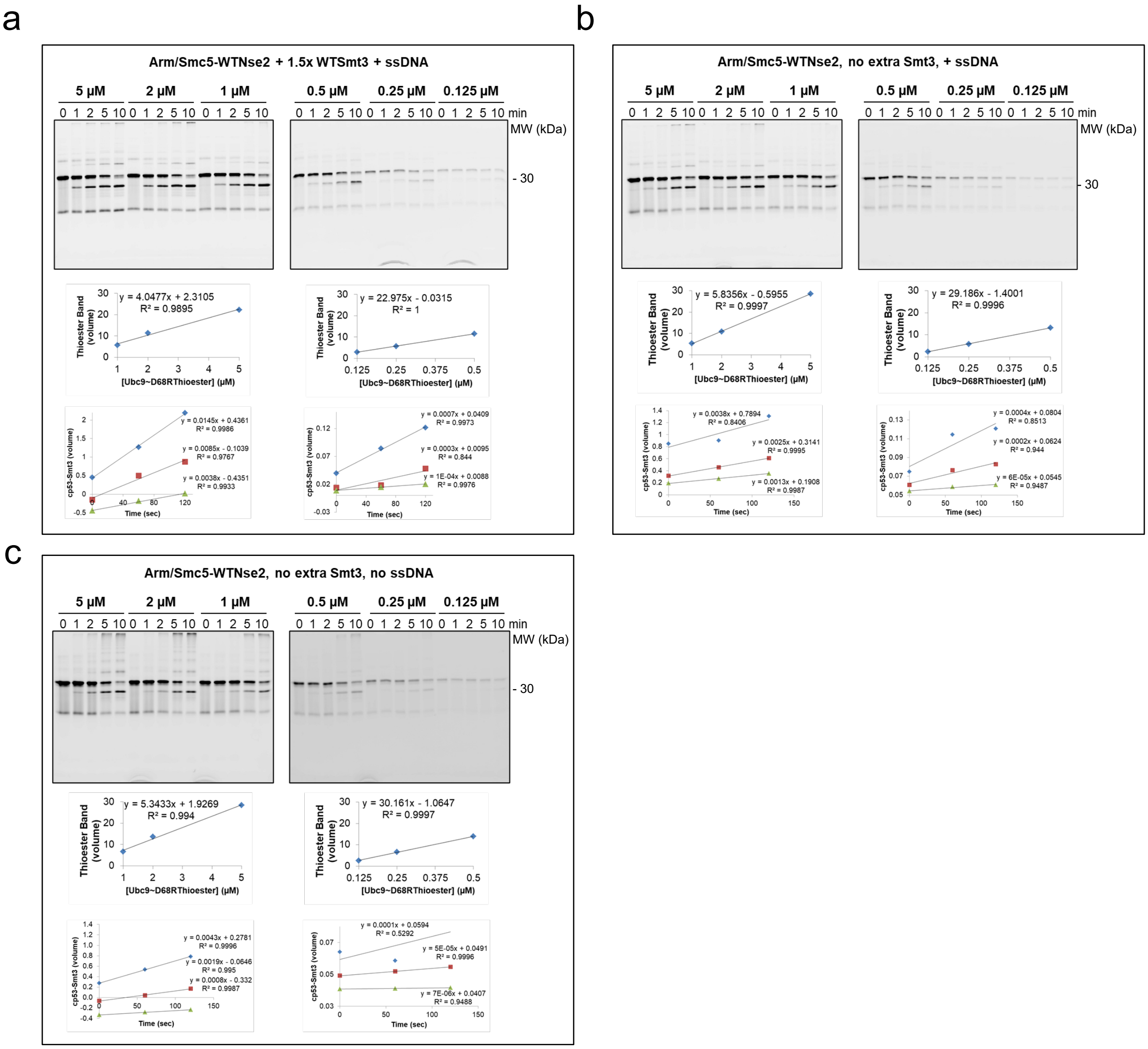


**Supplementary Figure 4. Raw data of SUMOylation reactions of Nse2/Smc5 complex using purified Ubc9~$^{D68R}$Smt3 thioester in the presence of extra wild-type or ssDNA. a-c** Representative SDS-PAGEs of the single-turnover SUMOylation reactions using increasing concentrations of purified Ubc9~$^{D68R}$Smt3-Alexa488 thioester, $_{c}$p53, yeast Nse2/Smc5 complex in the presence or absence of non-conjugatable extra wild-type Smt3 or 50 nt ssDNA. Line graph showing results of band densitometry measurements.

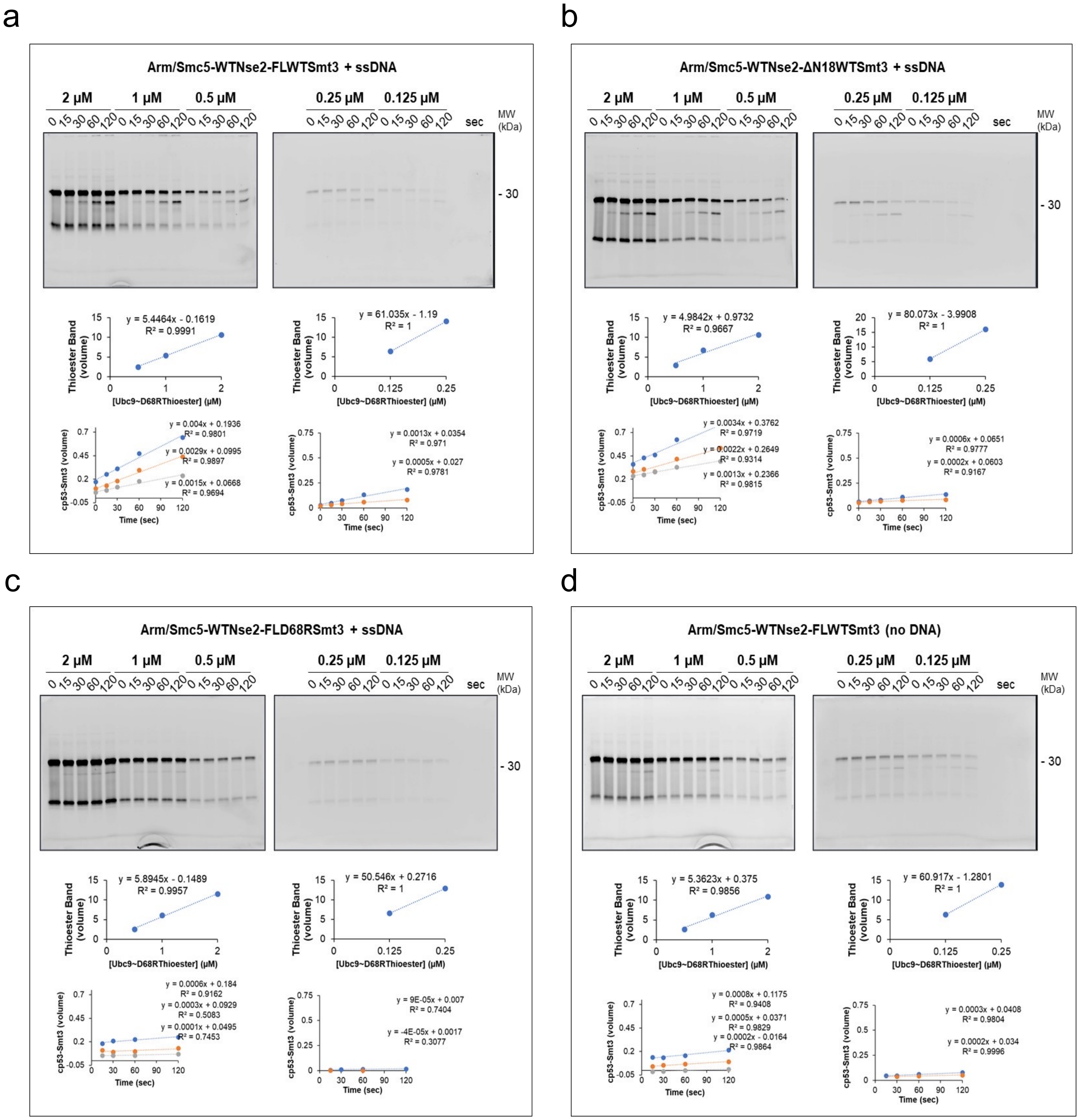


**Supplementary Figure 5. Raw data of SUMOylation reactions of different Nse2-Smt3/Smc5 fusion complexes using purified Ubc9~$^{D68R}$Smt3 thioester.** A-d Representative SDS-PAGEs of the single-turnover SUMOylation reactions using increasing concentrations of purified Ubc9~$^{D68R}$Smt3-Alexa488 thioester, $_{c}$p53, yeast Nse2-Smt3/Smc5 fusion complexes (FLWT, ΔN18WT, FLD68R) in the presence or absence of 50 nt ssDNA. Line graph showing results of band densitometry measurements.

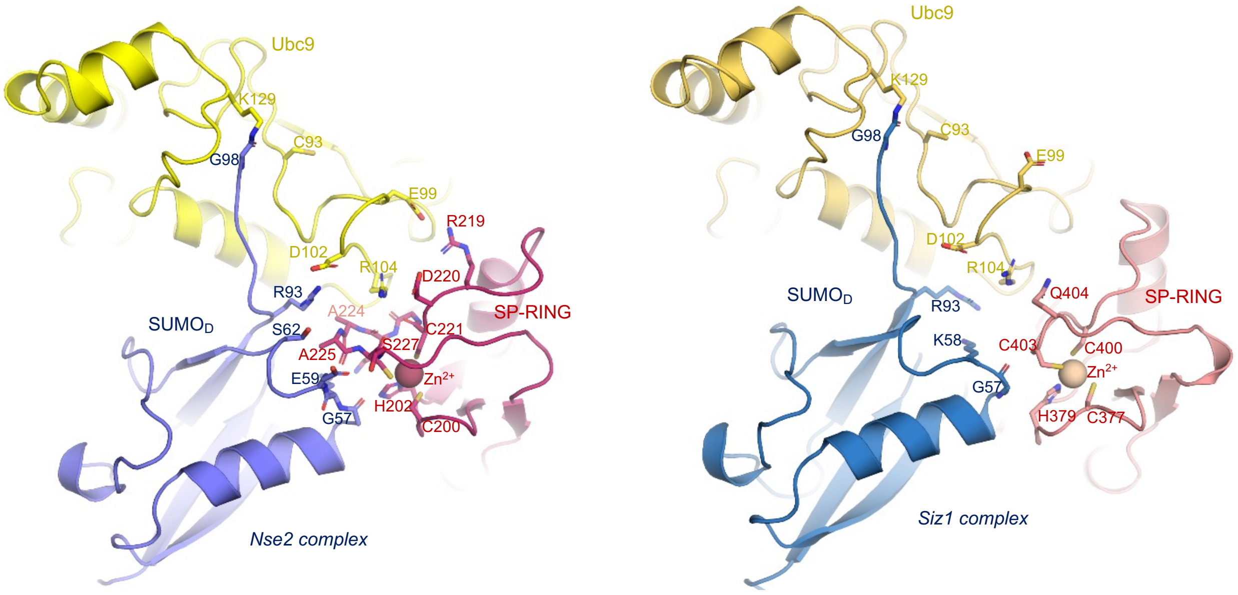


**Supplementary Figure 6. Binding interfaces of Smt3~Ubc9 and SP-RING domains of yeast Nse2 and Siz1.** Side-views of the structures of Nse2/Smc5 and Siz1 in complex with Ubc9~Smt3 thioester mimetic (PDBs 7P47 and 5JNE), revealing that the SP-RING in Nse2 uses similar contacts as ubiquitin RING E3s to bind SUMO$_D$, such interaction is not observed in the SP-RING of Siz1.

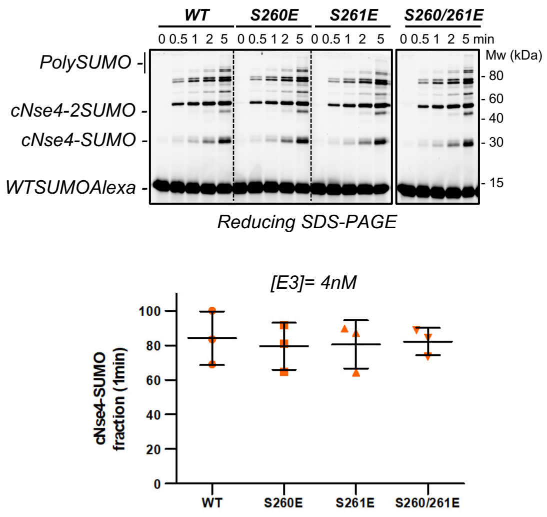


**Supplementary Figure 7. Substitution of Ser260 and Ser261 for negatively charged residues nearby SIM2.** Multiple-turnover SUMOylation reactions of yeast Nse2/Arm-Smc5 complex (wild type and mutants) using cNse4 substrate. Error bars showing that point mutations of serine to glutamic acid nearby SIM2 does not increase E3 activity. Data values represent the mean ±SD, n = 3 technical replicates. Significance was measured by a two-tailed unpaired t-test relative to wild-type. All data were analyzed with a 95% confidence interval. Exact *P* values from the left to right: 0.771, 0.778, 0.848. Source data are provided as a Source Data file.
